# Noether symmetries in Gauss–Bonnet-teleparallel cosmology

**DOI:** 10.1140/epjc/s10052-016-4491-0

**Published:** 2016-11-18

**Authors:** Salvatore Capozziello, Mariafelicia De Laurentis, Konstantinos F. Dialektopoulos

**Affiliations:** 1 Dipartimento di Fisica “E. Pancini”, Universita’ di Napoli“Federico II”, Complesso Universitario di Monte S. Angelo, Edificio G, Via Cinthia, 80126 Napoli, Italy; 2 INFN Sezione di Napoli, Complesso Universitario di Monte S. Angelo, Edificio G, Via Cinthia, 80126 Napoli, Italy; 3 Gran Sasso Science Institute (INFN), Via F. Crispi 7, 67100 L’ Aquila, Italy; 4 Tomsk State Pedagogical University, 634061 Tomsk, Russia; 5 Institute for Theoretical Physics, Goethe University, Max-von-Laue-Str. 1, 60438 Frankfurt, Germany; 6 Laboratory of Theoretical Cosmology, Tomsk State University of Control Systems and Radioelectronics (TUSUR), 634050 Tomsk, Russia

## Abstract

A generalized teleparallel cosmological model, $f(T_\mathcal {G},T)$, containing the torsion scalar *T* and the teleparallel counterpart of the Gauss–Bonnet topological invariant $T_{\mathcal {G}}$, is studied in the framework of the Noether symmetry approach. As $f(\mathcal {G}, R)$ gravity, where $\mathcal {G}$ is the Gauss–Bonnet topological invariant and *R* is the Ricci curvature scalar, exhausts all the curvature information that one can construct from the Riemann tensor, in the same way, $f(T_\mathcal {G},T)$ contains all the possible information directly related to the torsion tensor. In this paper, we discuss how the Noether symmetry approach allows one to fix the form of the function $f(T_\mathcal {G},T)$ and to derive exact cosmological solutions.

## Introduction

Extended theories of gravity are semi-classical approaches where the effective gravitational Lagrangian is modified, with respect to the Hilbert–Einstein one, by considering higher-order terms of curvature invariants, torsion tensor, derivatives of curvature invariants and scalar fields (see for example [1–4]). In particular, taking into account the Ricci, Riemann, and Weyl invariants, one can construct terms like $R^2,$ $R^{\mu \nu }R_{\mu \nu },$ $R^{\mu \nu \delta \sigma }R_{\mu \nu \delta \sigma }$, $W^{\mu \nu \delta \sigma }W_{\mu \nu \delta \sigma },$ that give rise to fourth-order theories in the metric formalism [5, 6]. Considering minimally or nonminimally coupled scalar fields to the geometry, we deal with scalar–tensor theories of gravity [7, 8]. Considering terms like $R\Box R,$ $R\Box ^k R$, we are dealing with higher-than fourth-order theories [9, 10]. *f*(*R*) gravity is the simplest class of these models where a generic function of the Ricci scalar *R* is considered. The interest for these extended models is related both to the problem of quantum gravity [2] and to the possibility to explain the accelerated expansion of the universe, as well as the structure formation, without invoking new particles in the matter/energy content of the universe [4–15]. In other words, the attempt is to address the dark side of the universe by changing the geometric sector and remaining unaltered the matter sources with respect to the Standard Model of particles. However, in the framework of this “geometric picture”, the debate is very broad involving the fundamental structures of gravitational interaction. Just to summarize some points, gravity could be described only by metric (in this case we deal with a metric approach), or by metric and connections (in this case, we are considering a metric-affine approach [16]), or by a purely affine approach [17]. Furthermore, dynamics could be related to curvature tensor, as in the original Einstein theory, to both curvature and torsion [18], or to torsion only, as in the so-called *teleparallel gravity* [19].

Starting from these original theories and motivations, one can build more complex Lagrangians, by using different combinations of curvature scalars and their derivatives, or topological invariants, such us the Gauss–Bonnet term, $\mathcal {G}$, as well as the torsion scalar *T*. Many theories have been proposed considering generic functions of such terms, like $f(\mathcal{G})$, *f*(*T*), $f(R, \mathcal{G})$, and *f*(*R*, *T*) [20–44]. However, the problem is *how many* and *what kind* of geometric invariants can be used, and, furthermore, what kind of physical information one can derive from them. For example, it is well known that *f*(*R*) gravity is the straightforward extension of the Hilbert–Einstein case which is $f(R)=R$, and *f*(*T*) is the extension of teleparallel gravity which is $f(T)=T$. However, if one wants to consider the whole information contained in the curvature invariants, one has to take into account also combinations of Riemann, Ricci, and Weyl tensors.^1^ As discussed in [26], assuming a $f(R, \mathcal{G}$ theory means to consider the whole curvature budget and then all the degrees of freedom related to curvature.

Assuming the teleparallel formalism, a $f(T_\mathcal{G},T)$ theory, where $T_\mathcal{G}$ is the torsional counterpart of the Gauss–Bonnet topological invariant, means to exhaust all the degrees of freedom related to torsion and then completely extend *f*(*T*) gravity. It is important to stress that the Gauss–Bonnet invariant derived from curvature differs from the same topological invariant derived from torsion in less than a total derivative, as we will show below, and then the dynamical information is the same in both representations. According to this result, the topological invariant allows a regularization of dynamics also in the teleparallel torsion picture (see [26, 45] for a discussion in the curvature representation).

The layout of the paper is the following. In Sect. 2, we sketch the basic ingredients of the $f(T_\mathcal{G},T)$ theory showing, in particular, the equivalence between $T_\mathcal{G}$ and $\mathcal G$. Section 3 is devoted to a derivation of the cosmological counterpart of the theory and to the derivation of the Noether symmetry. The specific forms of $f(T_\mathcal{G},T)$ function, selected by the Noether symmetry, are discussed in Sect. 4. Cosmological solutions are given in Sect. 5. Conclusions are drawn in Sect. 6.

## $f(T_\mathcal {G},T)$ gravity

In order to incorporate spin in a geometric description, as well as to bring gravity closer to its gauge formulation, people started, some years ago, to study torsion in gravity [18, 19]. An extensive review of torsional theories (teleparallel, Einstein–Cartan, metric-affine, etc.) is presented in [1]. If in considering the action of the teleparallel theory, i.e. in a curvature-free *vierbein* formulation, we replace the torsion scalar, *T*, with a generic function of it, we obtain the so-called *f*(*T*) gravity [46–49],

In this paper, we will study a theory whose Lagrangian is a generic function of the Gauss–Bonnet-teleparallel term, $T_\mathcal {G}$ and the torsion scalar, *T*, i.e.

1 $$\begin{aligned} \mathcal{A}=\frac{1}{2\kappa }\int d^4 x\sqrt{-g}\left[ f(T_\mathcal {G},T)+\mathcal{L}_m\right] , \end{aligned}$$

which is a straightforward generalization of

2 $$\begin{aligned} \mathcal{A}=\frac{1}{2\kappa }\int d^4 x\sqrt{-g}\left[ f(T)+\mathcal{L}_m\right] , \end{aligned}$$

where $\mathcal{L}_m$ is the standard matter that, in the following considerations, we will discard. It is important to note that the field equations of *f*(*T*) gravity are of second order in the metric derivatives and thus simpler than those of *f*(*R*) gravity, which are of fourth order [1].

The metric determinant $\sqrt{-g}$ can be derived from the determinant of the vierbeins *h* as follows. We have

3 $$\begin{aligned} h^{\mu }{}_i h^i{}_{\nu } = \delta ^{\mu } {}_{\nu } \,,\,\, h^{\mu } {}_{i} h^{j} {}_{\mu } = \delta _i {}^j. \end{aligned}$$

The relation between metric and vierbeins is given by

4 $$\begin{aligned} g_{\mu \nu } = \eta _{ab} h^a {}_{\mu } h^{b} {}_{\nu }, \end{aligned}$$

where $\eta _{ab}$ is the flat Minkowski metric. Finally, it is $|h|\equiv$ det$\left( h^i_\mu \right) =\sqrt{-g}$. More details on how the two formalisms are related can be found in [29].

The torsion scalar is given by the contraction

5 $$\begin{aligned} T= S^{\mu \nu } {}_{\rho } T^{\rho } {}_{\mu \nu } \end{aligned}$$

where

6 $$\begin{aligned} S_{\rho } {}^{\mu \nu }= & {} \frac{1}{2}\left( K^{\mu \nu }{}_{\rho } + \delta ^{\mu } {}_{\rho } T^{\sigma \nu }{}_{\sigma } -\delta ^{\nu }{}_{\rho } T^{\sigma \mu }{}_{\sigma }\right) , \end{aligned}$$

7 $$\begin{aligned} K ^{\mu \nu } {}_{\rho }= & {} - \frac{1}{2} \left( T ^{\mu \nu } {}_{\rho } - T^{\nu \mu } {}_{\rho } - T_{\rho } {}^{\mu \nu }\right) ,\end{aligned}$$

8 $$\begin{aligned} T^{\alpha } {}_{\mu \nu }= & {} \Gamma ^{\alpha } {}_{\mu \nu } - \tilde{\Gamma } ^{\alpha } {}_{\mu \nu }, \end{aligned}$$

are, respectively, the superpotential, the contorsion tensor, the torsion tensor and $\tilde{\Gamma } ^{\alpha }{}_{\mu \nu }$ is the Weitzenböck connection.

Imposing the teleparallelism condition, the torsion scalar can be expressed as the sum of the Ricci scalar plus a total derivative term, i.e.

9 $$\begin{aligned} hT = - h\bar{R} + 2 \left( hT_{\nu }{}^{\nu \mu }\right) ,_{\mu }\;\; \Rightarrow \;\; T = - \bar{R} + 2 T_{\nu }{}^{\nu \mu },{}_{\mu }, \end{aligned}$$

where $\bar{R}$ here is the Ricci scalar corresponding to the Levi-Civita connection and *h*, as above, is the determinant of the metric. Following [28], the teleparallel equivalent of the Gauss–Bonnet topological invariant can be obtained:

10 $$\begin{aligned} h \mathcal {G} = h T_{\mathcal {G}} + \text {total derivative}, \end{aligned}$$

where the Gauss–Bonnet invariant, in terms of curvature, is

11 $$\begin{aligned} \mathcal {G} = R^2 -4 R_{\mu \nu }R^{\mu \nu }+ R_{\mu \nu \rho \sigma } R^{\mu \nu \rho \sigma }, \end{aligned}$$

and the teleparallel $T_{\mathcal {G}}$ invariant is given by

12 $$\begin{aligned} T_{\mathcal {G}}= & {} ( K^{\alpha _1} {}_{ea}K^{e \alpha _2}{}_{b}K^{\alpha _3} {}_{fc}K^{f\alpha _4}{}_{d} \nonumber \\&-\; 2 K^{\alpha _1 \alpha _2}{}_{a}K^{\alpha _3} {}_{eb} K^{e} {}_{fc}K^{f\alpha _4}{}_{d} \nonumber \\&+\; 2 K^{\alpha _1 \alpha _2}{}_{a}K^{\alpha _3}{}_{eb}K^{e\alpha _4}{}_{f}K^{f}{}_{cd} \nonumber \\&+\; 2 K^{\alpha _1 \alpha _2}{}_{a} K^{\alpha _3}{}_{eb} K^{e\alpha _4}{}_{c,d} ) \delta ^a {}_{\alpha _1} {}^b {}_{\alpha _2} {}^c {}_{\alpha _3} {}^d {}_{\alpha _4}. \end{aligned}$$

In a four dimensional spacetime, the term $T_{\mathcal {G}}$ is a topological invariant, constructed out of torsion and contorsion tensor.^2^ In order to simplify the notation, we will identify $T_{\mathcal {G}}$ with ${\mathcal {G}}$ from now on.

The field equations from the action (1) are then

13 $$\begin{aligned}&2{f_T}{\partial _\nu }\left( {h{h^\rho }_\kappa {S_\rho }^{\mu \nu }} \right) - 2h{f_T}{h^\gamma }_\kappa {S^{\rho \beta \mu }}{T_{\rho \beta \gamma }} \nonumber \\&\quad +\;2h{h^\rho }_\kappa {S_\rho }^{\mu \nu }{\partial _\nu }{f_T} + 4h{h_\kappa }^\nu R{R_{\mu \nu }}{f_{\mathcal {G}}} - \frac{1}{2}fh{h^\mu }_\kappa \nonumber \\&\quad +\;4h{h_\kappa }^\nu \left( {{g_{\mu \nu }}\square - {\nabla _\mu }{\nabla _\nu }} \right) \left( {R{f_{\mathcal {G}}}} \right) \nonumber \\&\quad +\;16h{h_\kappa }^\nu {\nabla ^\lambda }{\nabla _{(\mu }}\left( {{f_{\mathcal {G}}}{R_{\nu )\lambda }}} \right) \nonumber \\&\quad -\;8h{h_\kappa }^\nu {g_{\mu \nu }}{\nabla ^\alpha }{\nabla ^\beta }\left( {{f_{\mathcal {G}}}{R_{\alpha \beta }}} \right) - 8h{h_\kappa }^\nu \square \left( {{f_{\mathcal {G}}}{R_{\mu \nu }}} \right) \nonumber \\&\quad -\;16h{h_\kappa }^\nu {f_{\mathcal {G}}}{R_{\nu \alpha }}{R^\alpha }_\mu + 4h{h_\kappa }^\nu {f_{\mathcal {G}}}{R_\nu }^{\alpha \beta \gamma }{R_{\mu \alpha \beta \gamma }} \nonumber \\&\quad +\;8h{h_\kappa }^\nu {\nabla ^{(\rho }}{\nabla ^{\sigma )}}\left( {{f_{\mathcal {G}}}{R_{\mu \nu \rho \sigma }}} \right) = 0 \end{aligned}$$

where $$f_A=\partial f/\partial A$$ being $$A=T,\mathcal {G}$$.

In the discussion below, we will consider the Friedmann–Robertson–Walker (FRW) cosmology related to $f(T_\mathcal{G},T)$, i.e. $f(\mathcal{G},T)$, and we search for Noether symmetries in order to fix the form of the function *f* and to derive exact cosmological solutions.

## Searching for Noether symmetries

Let us consider a a spatially flat FRW cosmology defined by the line element

14 $$\begin{aligned} \mathrm{d}s^2 = -\mathrm{d}t^2 + a^2(t) (\mathrm{d}x^2 + \mathrm{d}y^2 + \mathrm{d}z^2), \end{aligned}$$

from which we can express the teleparallel Gauss–Bonnet term as a function of the scale factor *a*(*t*) [50]

15 $$\begin{aligned} T_\mathcal {G}= \mathcal {G}= 24 \left[ \frac{\dot{a}^2 (t)\ddot{a}(t)}{a(t)^3}\right] . \end{aligned}$$

As said above, we can discard the total derivative term (see also [30]) The torsion scalar is

16 $$\begin{aligned} T = - 6\left[ \frac{\dot{a}^2(t)}{a^2(t)}\right] . \end{aligned}$$

We can reduce (1) to a canonical point-like action by using the Lagrange multipliers as

17 $$\begin{aligned} \mathcal {A} = \frac{1}{2 \kappa }\int \mathrm{d}t \left[ a^3 f(\mathcal {G},T)- \lambda _1 \left( \mathcal {G} - \bar{\mathcal {G}}\right) - \lambda _2 \left( T - \bar{T}\right) \right] ,\nonumber \\ \end{aligned}$$

where $\bar{\mathcal {G}}$ and $\bar{T}$ are the Gauss–Bonnet term and the torsion scalar expressed by (15) and (16). The Lagrange multipliers are given by $\lambda _1 = a^3 \partial _{\mathcal {G}}f =a^3 f_{\mathcal {G}}$ and $\lambda _2 = a^3 \partial _{T}f = a^3 f_T$ and are obtained by varying the action with respect to $\mathcal {G}$ and *T*, respectively. We can rewrite the action (17) as

18 $$\begin{aligned} \mathcal {A}= & {} \int \frac{\mathrm{d}t a^3}{2\kappa } \left[ \phantom {\left( T+6 \frac{\dot{a^2}}{a^2}\right) }f(\mathcal {G},T) - f_{\mathcal {G}} \left( \mathcal {G} - \frac{24 \dot{a} ^2 \ddot{a}}{a^3}\right) \right. \nonumber \\&\left. -\;f_T\left( T+6 \frac{\dot{a^2}}{a^2}\right) \right] \end{aligned}$$

and, discarding total derivative terms, the final Lagrangian is

19 $$\begin{aligned} \mathcal {L} = a^3 \left( f - \mathcal {G} f_{\mathcal {G}} - T f_T\right) - 8 \dot{a}^3 \left( \dot{\mathcal {G}} f_{\mathcal {G}\mathcal {G}}+\dot{T}f_{\mathcal {G}T}\right) - 6 f_T a \dot{a}^2.\nonumber \\ \end{aligned}$$

This is a point-like, canonical Lagrangian whose configuration space is ${\mathbb {Q}}= \{a,\mathcal {G},T\}$ and tangent space is ${\mathbb {TQ}} = \{a, \dot{a},\mathcal {G},$ $\dot{\mathcal {G}},T,\dot{T}\}$. The Euler–Lagrange equations for $a,\,\mathcal {G}$ and *T* are, respectively,

20 $$\begin{aligned}&{a^2}\left( {f - \mathcal {G}{f_{\mathcal {G}}} - T{f_T}} \right) + 2{f_T}{{\dot{a}}^2} + 16\dot{a}\ddot{a}{{\dot{f}}_{\mathcal {G}}} + 8{{\dot{a}}^2}{{\ddot{f}}_{\mathcal {G}}} \nonumber \\&\quad +\;4{{\dot{f}}_T}a\dot{a} + 4{f_T}a\ddot{a} =0, \end{aligned}$$

21 $$\begin{aligned}&\left( {{a^3}\mathcal {G} - 24{{\dot{a}}^2}\ddot{a}} \right) {f_{\mathcal {G}\mathcal {G}}} + \left( {{a^3}T + 6a{{\dot{a}}^2}} \right) {f_{T\mathcal {G}}} = 0, \end{aligned}$$

22 $$\begin{aligned}&\left( {{a^2}T - 6{{\dot{a}}^2}} \right) a{f_{TT}} - \left( {{a^3}\mathcal {G} - 24{{\dot{a}}^2}\ddot{a}} \right) {f_{\mathcal {G}T}}=0. \end{aligned}$$

As expected, for $f_{\mathcal {G}\mathcal {G}}\ne 0$ and $f_{\mathcal {G}T}\ne 0$, we obtain, from (21) and (22), Eqs. (15) and (16) for the Gauss–Bonnet term and the torsion scalar. The energy condition $E_{\mathcal {L}}=0$, associated with Lagrangian (19), is

$$\begin{aligned} E_\mathcal{L}= \frac{\partial \mathcal{L}}{\partial {\dot{a}}}{\dot{a}}+\frac{\partial \mathcal{L}}{\partial {\dot{T}}}{\dot{T}}+\frac{\partial \mathcal{L}}{\partial {\dot{\mathcal{G}}}} {\dot{\mathcal{G}}}-\mathcal{L}&=0, \end{aligned}$$

corresponding to the 00-Einstein equation

23 $$\begin{aligned} 24 \dot{a} ^3 {\dot{f}}_{\mathcal {G}} + 6 f_T a \dot{a} ^2 + a^3 \left( f- \mathcal {G} f_{\mathcal {G}} - T f_T\right) = 0. \end{aligned}$$

Alternatively, the system (20)–(23) can be derived from the field equation (13).

Let us now use the Noether symmetry approach [31] to find possible symmetries for the dynamical system given by the Lagrangian (19).

In general, a Lagrangian admits a Noether symmetry if its Lie derivative, along a vector field *X*, vanishes^3^

24 $$\begin{aligned} L_{X}\mathcal {L} = 0 \Rightarrow X\mathcal {L} = 0. \end{aligned}$$

Alternatively, the existence of a symmetry depends on the existence of a vector (a “complete lift”), which is defined on the tangent space of the Lagrangian, i.e.

25 $$\begin{aligned} X = \alpha ^i (q)\frac{\partial }{\partial q^i}+\frac{d\alpha ^i (q)}{\mathrm{d}t}\frac{\partial }{\partial \dot{q}^i}, \end{aligned}$$

$q^i$ being the configuration variables, $\dot{q}^i$ the generalized velocities, and $\alpha ^i(q^j)$ the components of the Noether vector. In our case, the Lagrangian admits three degrees of freedom and then the symmetry generator (25) reads

26 $$\begin{aligned} X=\alpha \frac{\partial }{\partial a}+ \beta \frac{\partial }{\partial \mathcal{G}}+\gamma \frac{\partial }{\partial T}+{\dot{\alpha }} \frac{\partial }{\partial \dot{a}}+ {\dot{\beta }}\frac{\partial }{\partial \dot{\mathcal{G}}}+\dot{\gamma }\frac{\partial }{\partial \dot{T}}. \end{aligned}$$

The system derived from Eq. (24) consists of 10 partial differential equations (see [31] for details), for $\alpha ,$ $\beta ,$ $\gamma$, and $f(\mathcal {G},T)$. It is overdetermined and, if solved, it allows us to determine the components of the Noether vector and the form of $f(\mathcal {G},T)$. It is

27 $$\begin{aligned}&\partial _a \beta f_{\mathcal {G}\mathcal {G}} + \partial _a \gamma f_{\mathcal {G}T} = 0 \end{aligned}$$

28 $$\begin{aligned}&\beta f_{\mathcal {G}\mathcal {G}\mathcal {G}} + \gamma f_{\mathcal {G}\mathcal {G}T}+ 3 \partial _a \alpha f_{\mathcal {G}\mathcal {G}}+\partial _{\mathcal {G}} \beta f_{\mathcal {G}\mathcal {G}} + \partial _{\mathcal {G}}\gamma f_{\mathcal {G}T} = 0, \end{aligned}$$

29 $$\begin{aligned}&\beta f_{\mathcal {G}T\mathcal {G}} + \gamma f_{\mathcal {G}TT} + 3 \partial _a \alpha f_{\mathcal {G}T} + \partial _T \beta f_{\mathcal {G}\mathcal {G}} + \partial _T \gamma f_{\mathcal {G}T} = 0, \end{aligned}$$

30 $$\begin{aligned}&\alpha f_T + \beta f_{T\mathcal {G}}a + \gamma f_{TT} a + 2 f_T a \partial _a \alpha = 0, \end{aligned}$$

31 $$\begin{aligned}&a f_T \partial _{\mathcal {G}}\alpha = 0, \end{aligned}$$

32 $$\begin{aligned}&a f_T \partial _{T}\alpha = 0, \end{aligned}$$

33 $$\begin{aligned}&f_{\mathcal {G}\mathcal {G}} \partial _{\mathcal {G}}\alpha = 0, \end{aligned}$$

34 $$\begin{aligned}&f_{\mathcal {G}T}\partial _T \alpha = 0,\end{aligned}$$

35 $$\begin{aligned}&\partial _{\mathcal {G}} \alpha f_{\mathcal {G}T} + \partial _T \alpha f_{\mathcal {G}\mathcal {G}} = 0, \end{aligned}$$

36 $$\begin{aligned}&3 \alpha \left( f- \mathcal {G}f_{\mathcal {G}}-Tf_T\right) - a \beta \left( \mathcal {G} f_{\mathcal {G}\mathcal {G}}+T f_{T\mathcal {G}} \right) \nonumber \\&\quad -\;a \gamma \left( \mathcal {G} f_{\mathcal {G}T}+T f_{TT} \right) = 0. \end{aligned}$$

Clearly, it being a system of partial differential equations, a theorem of existence and unicity for the solutions does not hold. However, if only one of the functions $\alpha ,\beta , \gamma$ is different from zero, a Noether symmetry exists. Below, we will show that the existence of the symmetry selects the form of the function $f(\mathcal{G},T)$ and allows one to get exact solutions for the dynamical system (20)–(23).

## Selecting the form of $f(\mathcal {G},T)$ by symmetries

In order to solve the above system, we have to make some assumptions. There are two ways to look for solutions: the first is to assume specific families of $f(\mathcal {G},T)$ and derive symmetries accordingly, i.e. find the components of the symmetry vector. The second approach consists in imposing a specific form for the symmetry vector and then finding the form of $f(\mathcal {G},T)$. However, in the second case, the chosen functions $\alpha , \beta , \gamma$ must be a solution of the system (27)–(36). This approach is straightforward and more mathematically consistent. It consists in reducing one of the above equations, e.g. Eq. (36), to a differential equation for $f(\mathcal {G},T)$, once the components of the Noether vector are assigned. As reported in [31] for the case of a scalar–tensor theory with only a scalar field $\phi$, by assigning $\alpha$ and $\beta$, it is possible^4^ to derive a constraint differential equation for the coupling $F(\phi )$ and then for the potential $V(\phi )$. The general solution has to be discarded since it is an implicit function of $F(\phi )$ without a physical meaning and then useless. On the other hand, the particular solution $F(\phi )=\xi \phi ^2$ is physically meaningful and then can be used to find relevant cosmological solutions (see [31] for details). In the present case, the constraint for $f(\mathcal {G},T)$ is a differential equation in two variables. Achieving general solutions does not give physically relevant and workable models.

To obtain physically reliable models, the first route is more convenient. In this preliminary paper, we will adopt this strategy to find solutions choosing classes of $f(\mathcal {G},T)$ function.

### The case $f(\mathcal {G},T)= g_0 \mathcal {G}^k + t_0 T^m$

We substitute this form of $f(\mathcal {G},T)$ in the system (27)–(36) and find that, for $k\ne 1$ and arbitrary *m*, the only possible Noether vector is the trivial one, $X=(0,0,0)$, which means that there is no symmetry. However, for $k = 1$ and arbitrary *m*, i.e. $f(\mathcal {G},T) = g_0 \mathcal {G}+t_0 T^m$, the vector assume the non-trivial form

37 $$\begin{aligned} X\equiv \left\{ \alpha _0 a^{1-\frac{3}{2 m}},\,\beta (a,\mathcal {G},T),\,-\frac{3 \alpha _0 T a^{-\frac{3}{2 m}}}{m}\right\} , \end{aligned}$$

with $\alpha _0$ being an arbitrary integration constant and any non singular $\beta$. This means that this theory admits a symmetry with the conserved quantity being

38 $$\begin{aligned} \Sigma _0=-12 \alpha _0 m t_0\left( \frac{\dot{a}}{a^{\frac{3}{2 m}-2}} \right) T^{m-1}, \end{aligned}$$

which coincides with the case $f(T)=t_0 T^m$ and then the contribution of the Gauss–Bonnet invariant is trivial.^5^ This is expected since, in a 4-dimensional manifold, the linear Gauss–Bonnet term is vanishing in the action and thus this model is not different from *f*(*T*) gravity.

### The case $f(\mathcal {G},T) = f_0 \mathcal {G}^k T^m$

In this case, the system (27)–(36) becomes slightly more complicated. As previously, we have two possible choices of the powers *k*, *m*. If $m\ne 1-k$, $f(\mathcal {G},T)$ reduces to pure *f*(*T*), i.e. we have to set $k=0$ and therefore we have the same symmetries as before. Nevertheless, if $m=1-k$, the model becomes $f(\mathcal {G},T)= f_0 \mathcal {G}^k T^{1-k}$ and it admits a Noether symmetry denoted by the vector

39 $$\begin{aligned} X=(0,\beta (a,\mathcal {G},T),\frac{T}{\mathcal {G}}\beta (a,\mathcal {G},T)), \end{aligned}$$

where $\beta$ is a non-singular function. It is interesting to point out the analogy with the curvature case, where the Noether symmetry approach selects the form $f(\mathcal {G},R)= f_0 \mathcal {G}^{1-k}R^{k}$ as discussed in [50]. In some sense, symmetries preserve the structure of gravitational theories independently of the teleparallel or metric formulation.^6^

## Cosmological solutions

Starting from the model $f(\mathcal {G},T)= f_0 \mathcal {G}^{k}T^{1-k}$, let us find cosmological solutions for any values of *k*. The Lagrangian (19) assumes the form

40 $$\begin{aligned} \mathcal {L}=f_0 (k-1) \dot{a}^2 \mathcal {G}^{k-2} T^{-k} \left[ 4 k \dot{a} \left( \mathcal {G} \dot{T} - T \dot{\mathcal {G}}\right) +3 a \mathcal {G}^2\right] .\nonumber \\ \end{aligned}$$

and the Euler–Lagrange equation for *a*(*t*) and the energy equation become

41 $$\begin{aligned}&2 k G^2 a' \left( 4 T a'' T'+a' \left( 2 T T''-2 k T'^2\right) +a T G'\right) \nonumber \\&\quad +\;4 k G T a' \left( a' \left( 2 (k-1) G' T'-T G''\right) -2 T a'' G'\right) \nonumber \\&\quad +\;G^3 \left( T \left( 2 a a''+a'^2\right) -2 k a a' T'\right) \nonumber \\&\quad -\;4 (k-2) k T^2 a'^2 G'^2 =0, \end{aligned}$$

42 $$\begin{aligned}&4 k a' \left( G T'-T G'\right) +a G^2 =0, \end{aligned}$$

while the other two, i.e. for $\mathcal {G}$ and *T*, give the Lagrange multipliers (15) and (16). If we substitute the constraints (15), (16) into Eq. (41), (42) we get

43 $$\begin{aligned}&2 a^2 \ddot{a}^4+k^2 \dot{a}^4 \ddot{a}^2-2 (k-1) k a \dddot{a} \dot{a}^3 \ddot{a}+4 k a^2 \dddot{a} \dot{a} \ddot{a}^2 \nonumber \\&\quad +\;a \dot{a}^2 \left( (k-2) k a \dddot{a}^2+(1-5 k) \ddot{a}^3+k a \ddddot{a} \ddot{a}\right) = 0, \end{aligned}$$

44 $$\begin{aligned}&a \ddot{a}^2 + k a \dddot{a} \dot{a} - k \dot{a}^2 \ddot{a} = 0. \end{aligned}$$

These general (for arbitrary $k\ne 1$) equations admit power-law solutions for the scale factor of the form

45 $$\begin{aligned} a(t) = a_0 t^s, \quad \text{ with } \; s=2k+1. \end{aligned}$$

It is easy to verify that the Gauss–Bonnet term and the torsion scalar behave asymptotically as $\mathcal {G} \sim 1/t^4$ and $T \sim 1/t^2,$ for any *k*.

From these considerations, it is easy to realize that any Friedmann-like, power-law solution can be achieved according to the value of *k*. For example, a dust solution is recovered for

46 $$\begin{aligned} a(t) = a_0 t^{2/3}, \quad \text{ with } \; k=-\frac{1}{6}; \end{aligned}$$

a radiation solution is for

47 $$\begin{aligned} a(t) = a_0 t^{1/2}, \quad \text{ with } \; k=-\frac{1}{4}; \end{aligned}$$

and a stiff matter one is for

48 $$\begin{aligned} a(t) = a_0 t^{1/3}, \quad \text{ with } \; k=-\frac{1}{3}. \end{aligned}$$

Power-law inflationary solutions are achieved, in general, for $s\ge 1$ and then $k\ge 0$.

Some comments are necessary at this point. Clearly, the above solutions cannot track the whole cosmological evolution but only limited phases. Achieving solutions capable of tracking completely the cosmic history could not be possible due to the fact that smooth, derivable solutions cannot account for phase transitions occurring during cosmic evolution. In [51], a detailed discussion is pursued in view of providing a unified description of the cosmic evolution ranging from early-times inflation to late-times acceleration. In particular, the authors consider models like

49 $$\begin{aligned} f(\mathcal {G}, T) = -T+\beta _1(T^2+\beta _2\mathcal {G})+\beta _3(T^2+\beta _4\mathcal {G})^2 \end{aligned}$$

where higher-order terms become significant at an early stage of evolution and lower-order terms are relevant today, according to the values of the parameters $\beta _i$. In such a way, the two accelerated phases of the cosmic evolutions can be achieved.

Considering the present discussion, this means that, in order to achieve cosmological models consistent with more than one phase of cosmic evolution, one need to consider more general classes of $f(\mathcal {G}, T)$ functions coming from Noether’s symmetries.

## Conclusions

In this paper, we discussed a theory of gravity where the interaction Lagrangian consists of a generic function $f(T_{\mathcal {G}},T)$ of the teleparallel Gauss–Bonnet topological invariant, $T_{\mathcal {G}}$, and the torsion scalar *T*. The physical reason for this approach is related to the fact that we want to study a theory where the full budget of torsional degrees of freedom are considered. Furthermore, it is easy to show that, from a dynamical point of view, the Gauss–Bonnet invariant, derived from curvature, $\mathcal G$, and the Gauss–Bonnet invariant, derived from torsion, $T_{\mathcal {G}}$, are equivalent and then we can consider a $f({\mathcal {G}},T)$ theory.

After these considerations, we searched for Noether symmetries in the cosmology derived from this model. We showed that specific forms of $f(\mathcal {G},T)$ admit symmetries and allow for the reduction of the dynamical system.

In particular, the class $f(\mathcal {G},T)= f_0 \mathcal {G}^kT^{1-k}$ results particularly interesting and, depending on the value of *k*, it is possible to achieve all the behaviors of standard cosmology as particular solutions.

Clearly, other cases can be considered and a systematic approach to find other solutions can be pursued. This will be the argument of a forthcoming paper where a general cosmological analysis will be developed.

## Footnotes

1 Clearly, this means that we are not considering higher-order derivative terms like $\Box R$, or derivative combinations of curvature invariants.

2 See Sect. 3 of [28] for the detailed derivation and discussion.

3 There exists a symmetry even if the Lagrangian changes by a total derivative term, but we will discuss the simplest case.

4 In this case, the configuration space is ${\mathbb {Q}}= \{a,\phi \}$ and the tangent space is ${\mathbb {TQ}} = \{a, \dot{a},\phi ,\dot{\phi }\}$. and then we need only two components $\alpha$ and $\beta$ for the Noether vector.

5 See Eqs. (455)–(457) in the review paper [1] and the discussion in [12].

6 Clearly also the case $f(\mathcal {G},T)= f_0 \mathcal {G}^{1-k}T^{k}$ gives a symmetry.
